# Osteoid Osteoma of the Capitate: A Case Report and Literature Review

**Published:** 2009-09-10

**Authors:** Abdullah Al Shaikhi, Jonah Hébert-Davies, Thomas Moser, Émilie Maillot, Alain M. Danino

**Affiliations:** Centre Hospitalier de l'Université de Montréal, 1560, rue Sherbrooke Est, Montréal, Quebec, Canada H2L 4M1

## Abstract

Osteoid osteoma is a benign bone tumor that rarely affects the carpal bones. Because of its nonspecific presentation in the wrist, it remains a diagnostic challenge. We report an unusual case of osteoid osteoma in the capitate where the diagnosis was delayed and the presentation was that of an aggressive natured lesion with considerable functional incapacitation. Diagnosis was made by computed tomographic scan of the wrist and surgical excision lead to a dramatic relief of symptoms.

Osteoid osteoma, a well-known benign tumor that was identified by Jaffe^1^ in 1935, produces pain, generally worse at night. It typically presents in the long bones but rarely occurs in the hand, affecting mostly the metacarpals and proximal phalanges whereas carpal bones involvement is fairly uncommon.^2^ Clinical presentations of these lesions are often uncharacteristic and can lead to misdiagnosis. We report here such a case in which initial diagnosis was missed and delayed treatment resulted in considerable functional loss for the patient.

## CASE REPORT

**Figure 1:**
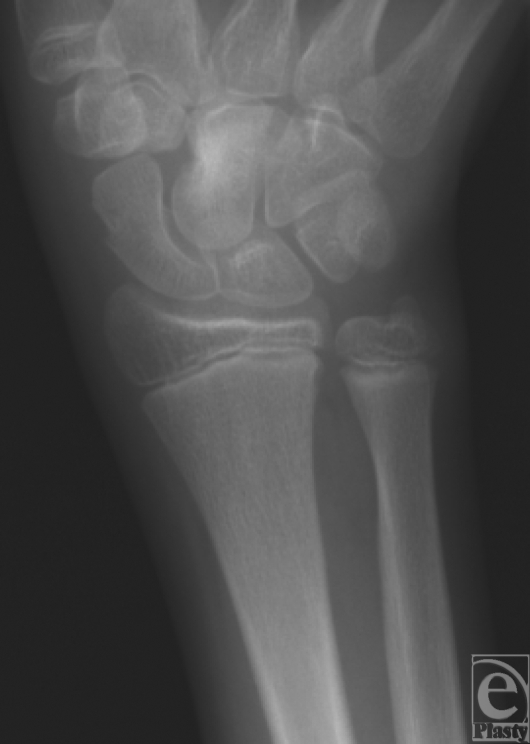
Original radiograph of the left wrist.

**Figure 2:**
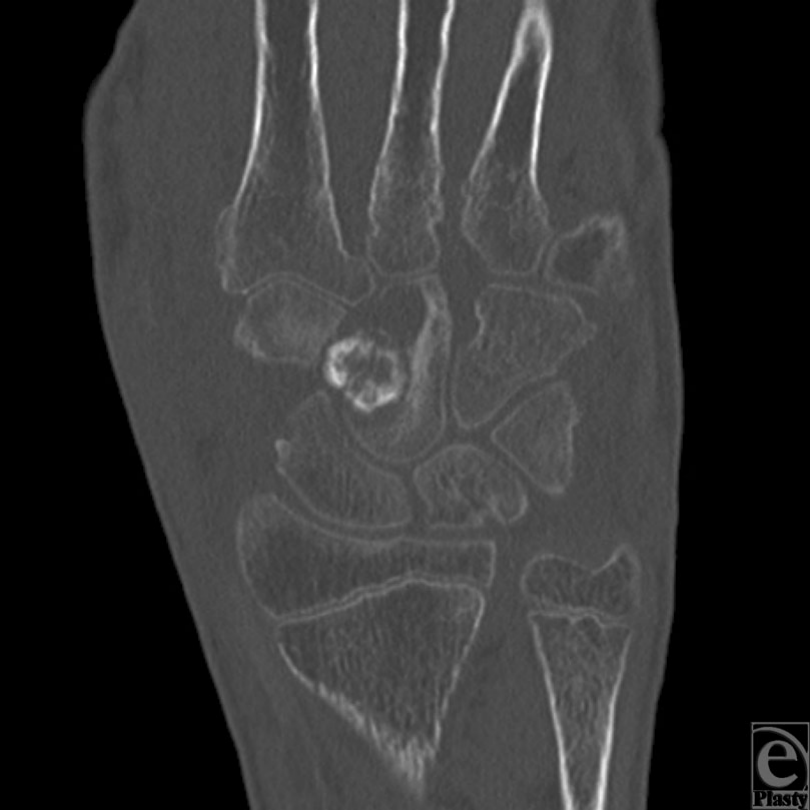
Preoperative computed tomographic scan. The nidus and destruction of the capitate are visible.

**Figure 3:**
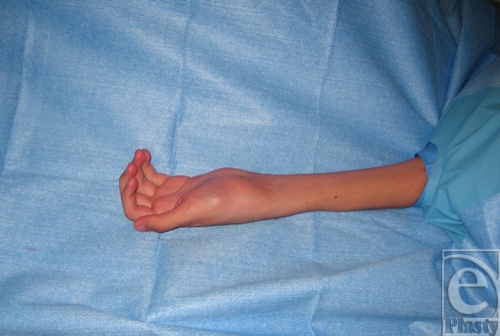
Gross deformation of the patient's left wrist seen preoperatively.

**Figure 4:**
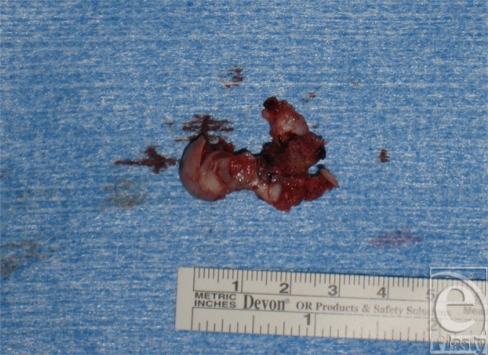
The excised capitate with visible destruction.

A 16-year-old right-handed male patient was referred to our tertiary university center for persistent left wrist pain. The patient had originally presented to another hospital nearly a year earlier after a fall on an outstretched hand. He was originally diagnosed with a left scaphoid fracture and treated with a forearm spica cast for 10 weeks. After removal, the pain persisted and a radiograph reported fracture consolidation. On follow-up visits, he continued to have severe pain, and despite aggressive physiotherapy, his wrist movement was very limited leading to muscular atrophy of his left hand and forearm. The patient was then referred to our facility with an impression of avascular necrosis of his scaphoid. On initial visit, the patient complained of constant pain and muscular atrophy in his left hand and forearm. On examination, marked atrophy of the left forearm and decreased muscular strength of the left hand were noted as well as hypersudation without other signs of complex regional pain syndrome. However, the patient was referred to pain management clinic for presumptive complex regional pain syndrome and further investigations were requested. On follow-up visit, the patient reported no improvement in symptoms and no longer used his left hand. Investigation demonstrated normal complete blood cell count and erythrocyte sedimentation rate. Hand radiograph demonstrated a lesion in the capitate (Fig 1). Computed tomographic (CT) scan of the hand demonstrated a lesion in the left capitate of more than 1 cm with necrosis and cortical destruction of adjacent bones and the possibility of aggressive transformation with a completely normal scaphoid (Fig 2). The patient underwent subsequent operation whereupon (Fig 3) exploration of the left wrist joint showed complete necrosis of the capitate and inflammatory degeneration of the hamate and trapezoid. A piecemeal excisional biopsy was performed on the capitate, hamate, and trapezoid of the left hand and the remaining gap was filled with antibiotic-containing bone cement (Fig 4). No frozen section biopsy was possible because of the nature of the specimen. There were no complications. Cultures performed during the excision gave negative results. The biopsy report identified an osteoid osteoma of the left capitate. This was later confirmed after review of the specimen by the pathology department at another center. Postoperatively, the patient reported a dramatic decrease in pain and an increase in range of motion of the left hand. After the final pathology report confirmed the diagnosis of osteoid osteoma, the patient underwent complete arthrodesis of the wrist with iliac crest bone graft, using the Haddad-Riordan technique.^3^ Following arthrodesis, the patient had a complete relief of pain, increasing range of motion, and complete consolidation and improvement in the shape of the forearm.

## DISCUSSION

Primary tumors of the carpal bones are extremely rare (0.16%); however, when they occur, they are generally benign (86%).^4^ Osteoid osteoma is the most common entity occurring in approximately 25% of cases.^4^ Generally, this lesion affects young individuals in the second and third decades of life, with the ratio of males affected at more than 2:1.^5^ It presents with increased night pain that responds well to nonsteroidal anti-inflammatory drugs.^2^ It is typically found in long bones and only rarely occurs in the wrist,^6^,^7^ where it is most commonly found in the scaphoid or capitate.^4^,^8^ A limited number of cases have been reported. Diagnosis of osteoid osteoma of the wrist is difficult because of the often vague nature of symptoms including spontaneous dull aching.^2^,^9^ Initial presentation can vary, among others, from extensor tendon tenosynovitis^10^,^11^ to carpal tunnel syndrome^12^ to, as in our case, suspected scaphoid fracture. Complex regional pain syndrome is also associated with the diagnosis.^13^ Differential diagnosis principally consists of cystic lesions and osteoblastoma.^14^,^15^ Plain radiographic diagnosis is regularly impossible because of the lack of typical findings (nidus) in the wrist^10^ and delayed appearance.^16^ Three-phase technetium-99m bone scan is fairly sensitive in detecting the lesion.^17^ Thin-slice CT scan is considered as the reference technique for the diagnosis of osteoid osteoma and is particularly useful when the nidus is hidden by complex anatomy. The nidus appears as a spherical or ovoid lucency containing variable central mineralization. Cortical thickening or solid periosteal reactions are associated with cortical osteoid osteoma.^18^ Subperiosteal osteoid osteoma is the most difficult to recognize. It presents either as a small cortical lucency without significant cortical thickening or as a focal cortical scalloping with a juxtacortical nidus. Intracapsular osteoid osteoma commonly manifests with regional osteoporosis.^19^ Computed tomographic scan and magnetic resonance imaging are better suited to enable diagnosis and also provide useful information for preoperative planning.^10^ Treatment of osteoid osteoma is generally considered to be en bloc excision.^20^ In case in which this is difficult, as is the case with the wrist and hand, curettage and excision with or without bone grafting have been deemed acceptable.^12^,^21^,^22^ The principal limitation of this technique is a higher rate of recurrence,^7^,^23^ which can be attributed to incomplete excision of the lesion. Also, prolonged use of anti-inflammatory treatment may lead to healing and CT-guided radiofrequency ablation has been used with high primary success rate (90%).^24^

## CONCLUSION

Osteoid osteoma of the wrist represents a diagnostic difficulty because of abnormal presentation and is often misdiagnosed. It may be responsible for considerable pain and functional incapacity. Appropriate imaging is necessary to make the diagnosis and includes CT scan, bone scan, and magnetic resonance imaging. Surgical treatment with en bloc resection is the preferred technique, though curettage is acceptable, and generally provides symptomatic relief.

## References

[1] Jaffe HL (1953). Osteoid-osteoma. Proc R Soc Med.

[2] Carroll RE (1953). Osteoid osteoma in the hand. J Bone Joint Surg Am.

[3] Haddad RJ, Riordan DC (1967). Arthrodesis of the wrist. A surgical technique. J Bone Joint Surg Am.

[4] Murray PM, Berger RA, Inwards CY (1999). Primary neoplasms of the carpal bones. J Hand Surg [Am].

[5] Freiberger RH, Loitman BS, Helpern M, Thompson TC (1959). Osteoid osteoma; a report on 80 cases. Am J Roentgenol Radium Ther Nucl Med.

[6] Kendrick JI, Evarts CM (1967). Osteoid-osteoma: a critical analysis of 40 tumors. Clin Orthop Relat Res.

[7] Golding JS (1954). The natural history of osteoid osteoma; with a report of twenty cases. J Bone Joint Surg [Br].

[8] de Smet L, Brys P, Fabry G, Baert A (1997). An unusual localisation and presentation of an osteoid osteoma. Acta Orthop Belg.

[9] Arazi M, Memik R, Yel M, Ogun TC (2001). Osteoid osteoma of the carpal bones. Arch Orthop Trauma Surg.

[10] Laffosse JM, Tricoire JL, Cantagrel A, Wagner A, Puget J (2006). Osteoid osteoma of the carpal bones. Two case reports. Joint Bone Spine.

[11] Fromm B, Martini A, Schmidt E (1992). Osteoid osteoma of the radial styloid mimicking stenosing tenosynovitis. A case report. J Hand Surg [Br].

[12] Arora J, McLauchlan J, Munro N (2003). Recurrent osteoid osteoma of the lunate: a case report and review of the literature. Hand Surg.

[13] Martorell F (1964). [Sudeck's atrophy due to osteoid osteoma of the carpal navicular]. Angiologia.

[14] Rosenfeld K, Bora FW, Lane JM (1973). Osteoid osteoma of the hamate. A case report and review of the literature. J Bone Joint Surg [Am].

[15] Fealy MJ, Lineaweaver W (1995). Intraosseous ganglion cyst of the scaphoid. Ann Plast Surg.

[16] Dockerty MB, Ghormley RK, Jackson AE (1951). Osteoid osteoma; a clinicopathologic study of 20 cases. Ann Surg.

[17] Marcuzzi A, Acciaro AL, Landi A (2002). Osteoid osteoma of the hand and wrist. J Hand Surg [Br].

[18] Herrlin K, Ekelund L, Lovdahl R, Persson B (1982). Computed tomography in suspected osteoid osteomas of tubular bones. Skeletal Radiol.

[19] Greenspan A (1993). Benign bone-forming lesions: osteoma, osteoid osteoma, and osteoblastoma. Clinical, imaging, pathologic, and differential considerations. Skeletal Radiol.

[20] Sim FH, Dahlin CD, Beabout JW (1975). Osteoid-osteoma: diagnostic problems. J Bone Joint Surg [Am].

[21] Girard J, Becquet E, Limousin M, Chantelot C, Fontaine C (2005). [Osteoma osteoid of the trapezoid bone: a case-report and review of the literature]. Chir Main.

[22] Bednar MS, Weiland AJ, Light TR (1995). Osteoid osteoma of the upper extremity. Hand Clin.

[23] Dunlop JA, Morton KS, Eliott GB (1970). Recurrent osteoid osteoma. Report of a case with a review of the literature. J Bone Joint Surg [Br].

[24] Cantwell CP, Obyrne J, Eustace S (2004). Current trends in treatment of osteoid osteoma with an emphasis on radiofrequency ablation. Eur Radiol.

